# Supplementary material for: Digital electronics in fibres enable fabric-based machine-learning inference
Source: Nat Commun. 2021 Jun 3;12:3317. doi: 10.1038/s41467-021-23628-5 (PMC8175338; doi:10.1038/s41467-021-23628-5)
Supplement: Supplementary file 1 — Supplementary Information [file 41467_2021_23628_MOESM1_ESM.pdf]

Supplementary Materials

Digital Electronics in Fibres enable Fabric-based Machine-Learning Inference

Gabriel Loke[1,2]†, Tural Khudiyev[2]†, Brian Wang[3], Stephanie Fu[3], Syamantak Payra[3], Yorai Shaoul[3], Johnny Fung[4], Ioannis Chatziveroglou[3], Pin-Wen Chou[5], Itamar Chinn[3], Wei Yan[2], Anna Gitelson-Kahn[6] , John Joannopoulos[7,8], Yoel Fink[1,2,4,7]*.

[1] Department of Materials Science and Engineering, Massachusetts Institute of Technology, Cambridge, MA 02139, USA

[2] Research Laboratory of Electronics, Massachusetts Institute of Technology, Cambridge, MA 02139, USA

[3] Department of Electrical Engineering and Computer Science, Massachusetts Institute of Technology, Cambridge, MA 02139, USA

[4] Department of Mechanical Engineering, Massachusetts Institute of Technology, Cambridge, MA 02139, USA

[5] Harrisburg University of Science and Technology, Harrisburg, PA 17101, USA

[6] Textile Department, Rhode Island School of Design, Providence, RI 02903, USA

[7] Institute for Soldier Nanotechnologies, Massachusetts Institute of Technology, Cambridge, MA 02139, USA

[8] Department of Physics, Massachusetts Institute of Technology, Cambridge, MA 02139, USA

*E-mail: yoel@mit.edu

†These authors contributed equally to this work

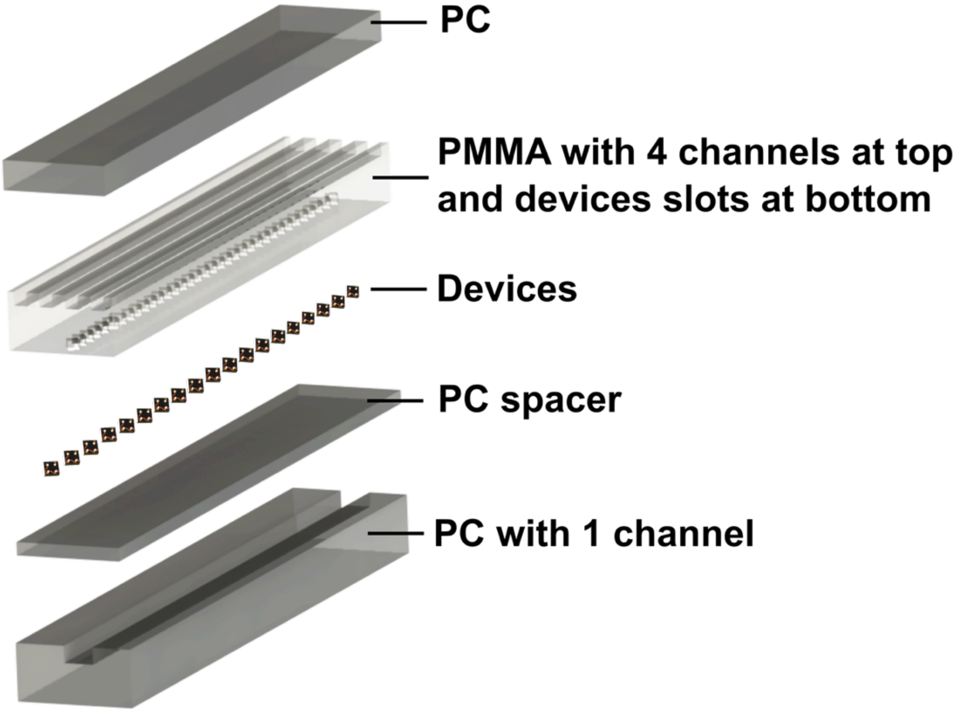

Supplementary Fig. 1: Digital Fibre Preform. Exploded view schematic of the preform, which consists of 4 polymeric slabs. The middle slab is made up of a soft polymer, PMMA, which contains milled slots at an angle of 26.56° as well as channels for the wire electrodes. Digital devices with square pad arrays are embedded into the slots. The rest of the slabs are made up of a harder polymer, polycarbonate (PC).

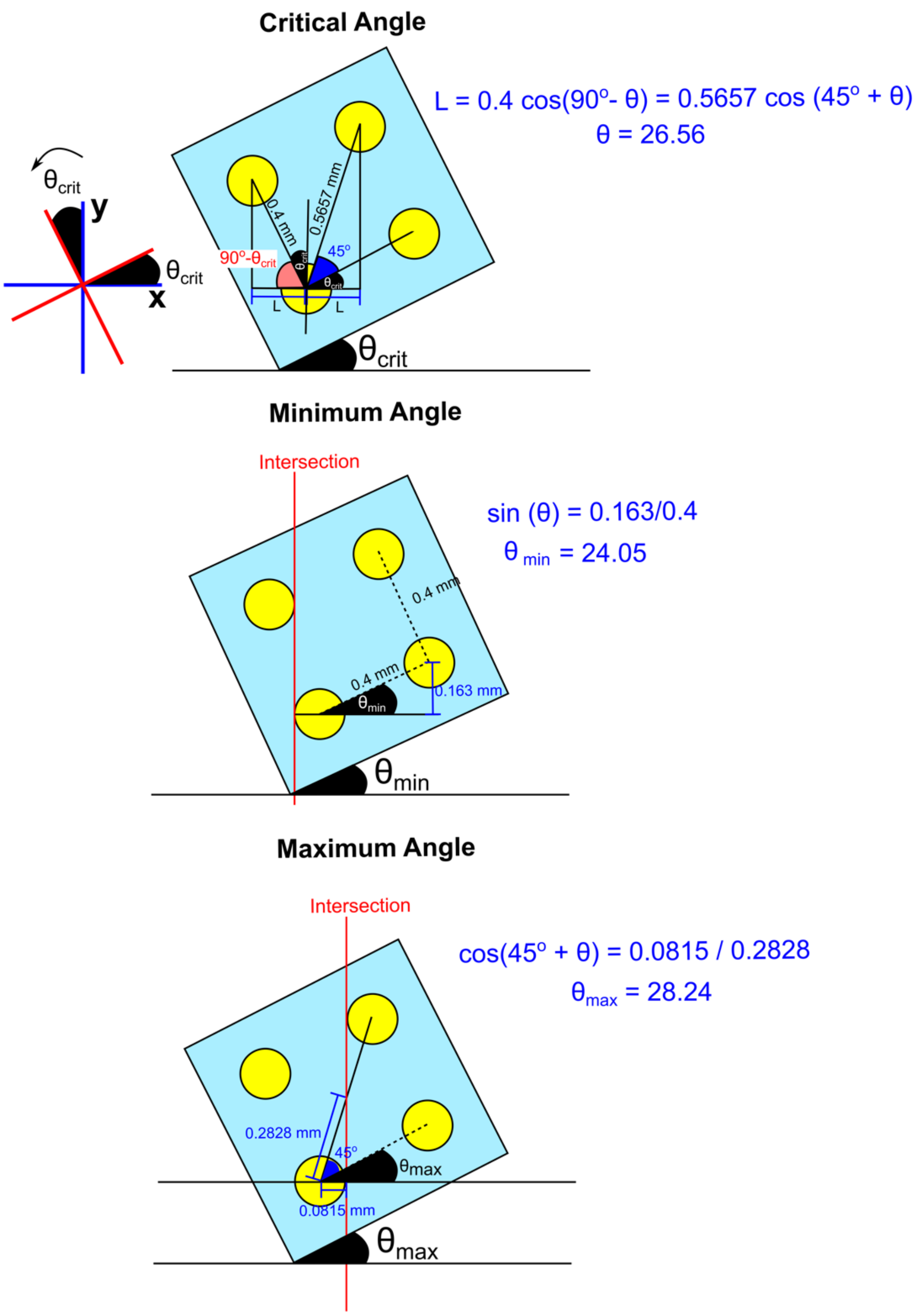

Supplementary Fig. 2: Calculation of the critical rotational angle of 26.56° for the embedded 4-pad digital chips. Note that this 4-pad configuration is an industrial standard. (left) The blue axes represent the x-axis and y-axis before rotation, and the red axes represent the x-axis and y-axis which are rotated. (Critical angle) To derive the critical rotational value, the device with the 4 circular pads (colored yellow) is rotated until there is an identical x-distance (segment L) between each pad. We then equate the geometric relations (equations represented on the right of each schematic) of the x-distances together to solve for the critical rotational angle. (Minimum angle) To find the minimum angle, the device is rotated until the edges of the leftmost 2 pads intersect (red line). We then solve the geometric relation to find for the minimum angle. (Maximum angle) To determine the maximum angle, the device is rotated until the edges of the inner 2 pads intersect (red line). We then solve the geometric relations (equation on the right) to obtain the maximum angle.

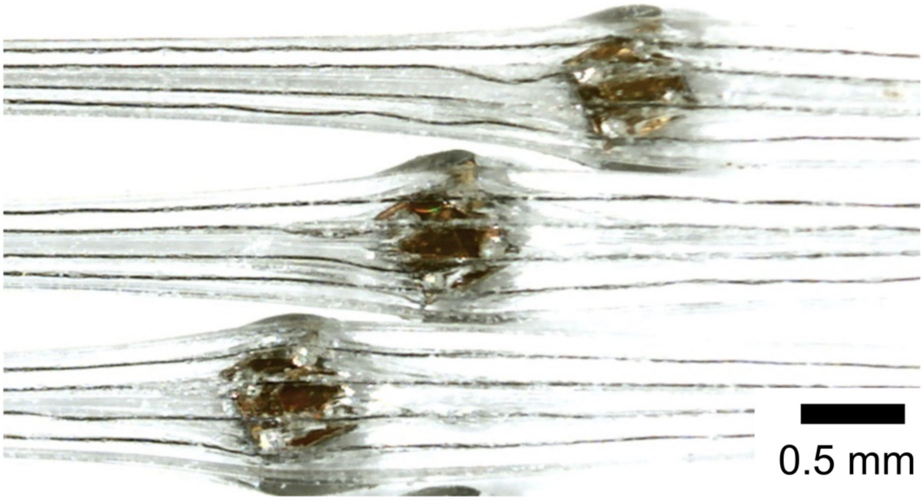

Supplementary Fig. 3: An array of digital fibres with embedded digital devices of the size ~ 500 µm by 500 µm. The four corner-positioned pads on each device is connected to four wires.

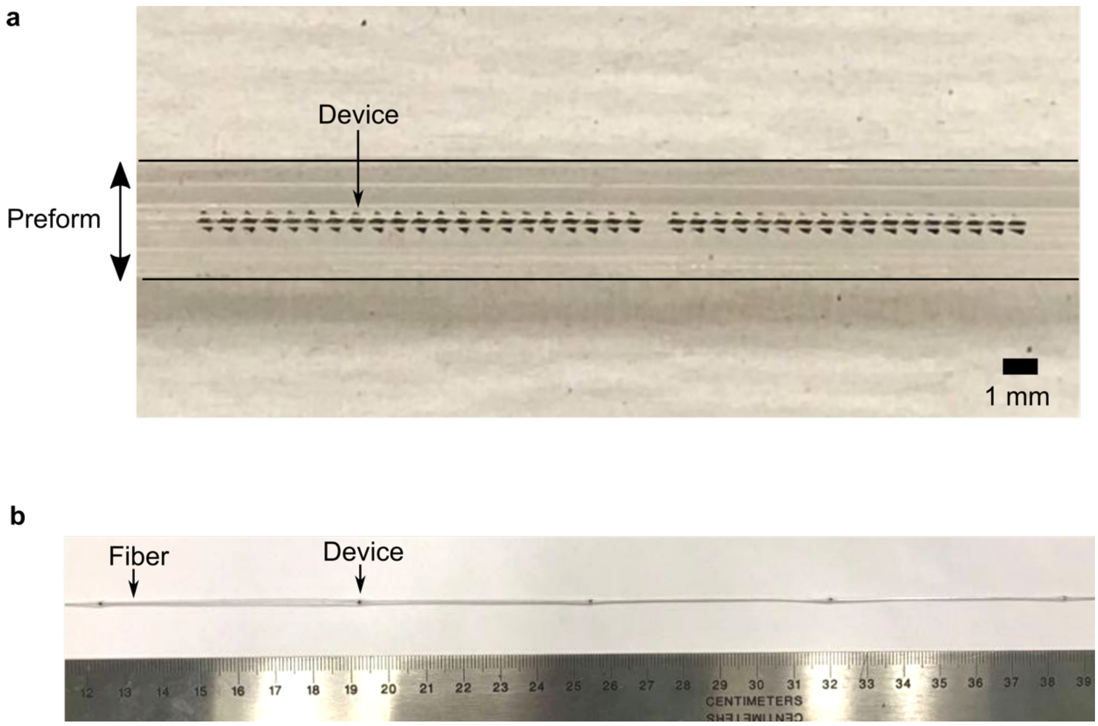

Supplementary Fig. 4: a, Optical magnification of the polymer preform with closely packed digital devices. There is a center-to-center distance of 0.65 mm between the devices. **b,** Photograph of a fibre with a draw-down ratio of 10, showing embedded devices spaced evenly at a distance of 6.5 cm. With this spacing, there are 15 devices per meter of fibre length.

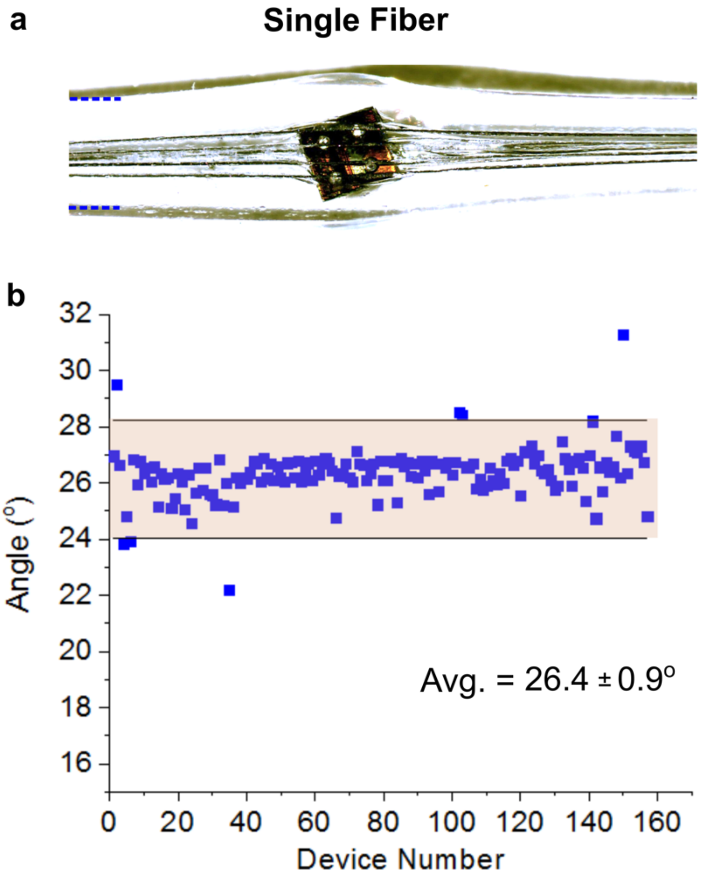

Supplementary Fig. 5: a, Magnified optical image of an individual fibre with its embedded device rotated to the angle of 26.56° and with wires connected to the four electrical contacts on the chip. **b,** Measurement of the angle across 157 in-fibre devices, with the average angle measured to be 26.4° and a standard deviation of 0.9°. The shaded region bounded by the upper and lower line represents the window of success of the rotational angles for the pads to remain non-crossing and non-intersecting along the fibre axis.

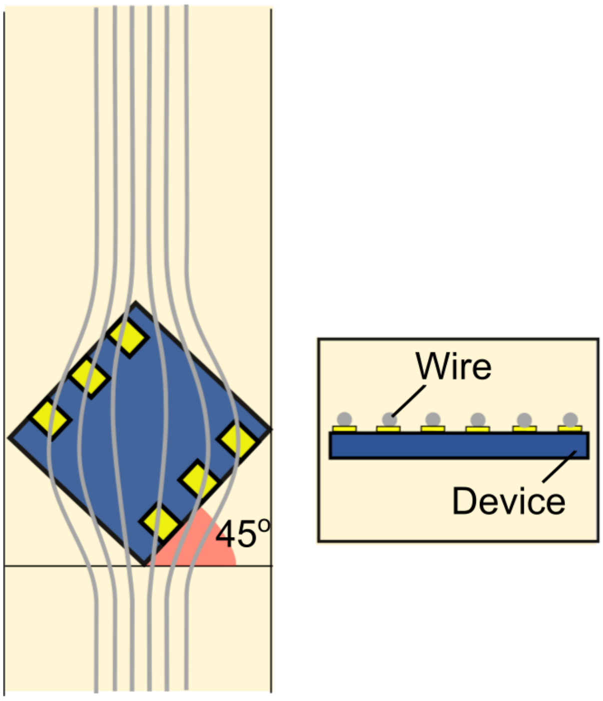

Supplementary Fig. 6: A potential scheme for in-fibre connection of devices with other common pad configurations, such as a 6-pad array. (left) Schematic of the rotated 6-pad device within the fibre, connected to 6 wires. (right) Schematic of the cross-section of the fibre at the device connections.

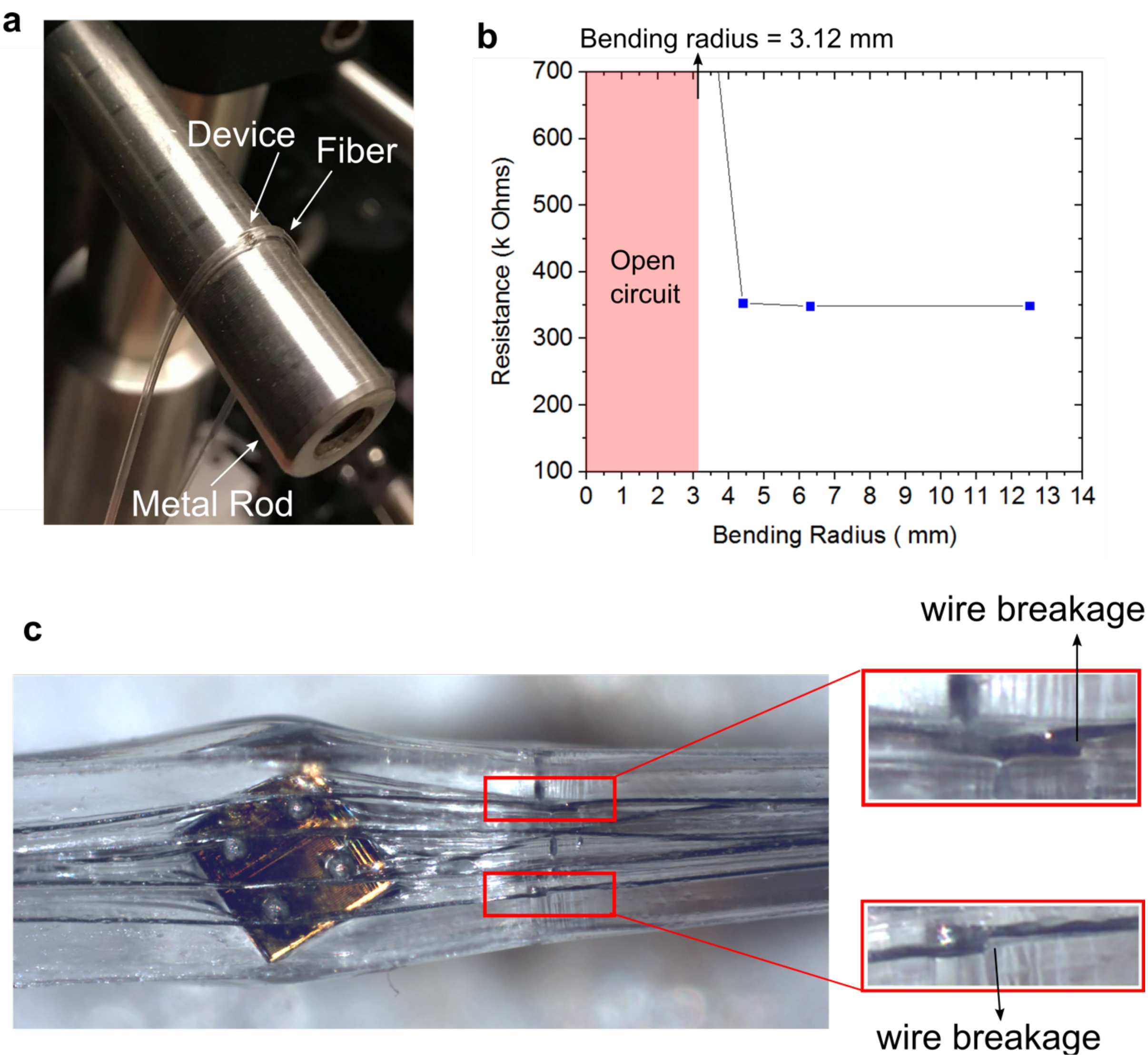

Supplementary Fig. 7: a, A photograph of the bending setup where the fibre is bend around stainless steel rods of different radius. **b,** Plot of resistance across two fibre wire electrodes for different bending radii. At large bending radius, the resistance remains in the range of kiloohms, signifying wire connections to the device. At small bending radius (threshold radius, r , being 3.12 mm), the W wires lost connections to the device, hence resulting in an overload in the resistance (signified by OL on the multimeter). **c,** Optical image of the device and wires in fibre after bending. On closer inspection, it is found that the overload in resistance is caused by the breakage of the W wires.

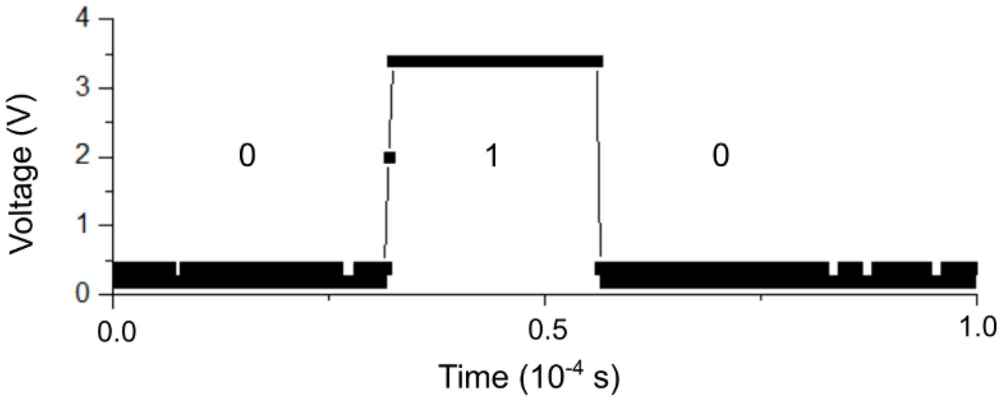

Supplementary Fig. 8: Minimum voltage requirement of 3.3V to write, store and read from the fibre memory.

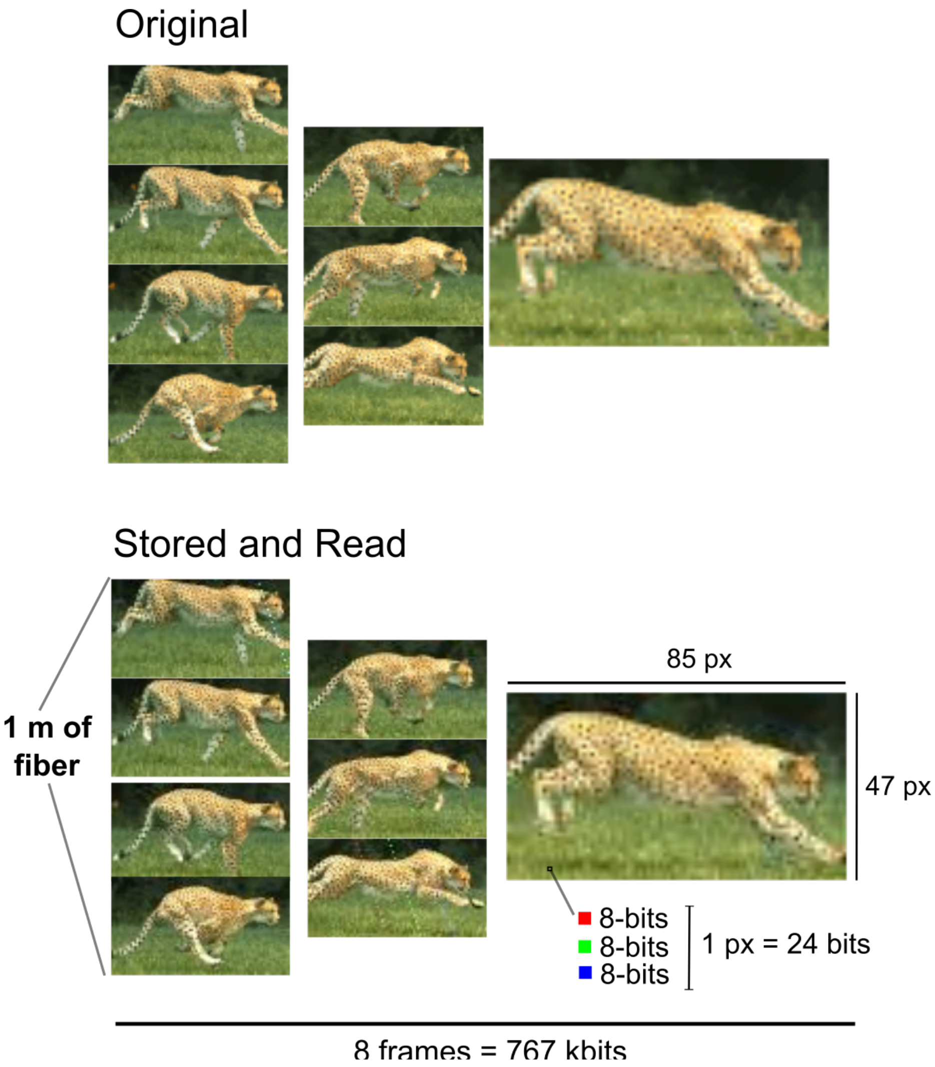

Supplementary Fig. 9: Within 1 meter of fibre, 767 kilobits of information can be stored. This allows for a short digital movie (8 frames of a cheetah running) to be stored within a single, one meter-long fibre. The frames at the top are the original pictures that were written into the fibre. The fibre was then stored for 2 months without connection to a power source. The frames at the bottom depict the output frames that were read from the fibre after storage for 2 months, showing long-term stability of in-fibre storage. The video file is downloaded at https://commons.wikimedia.org/wiki/File:Cheetahs_on_the_Edge_(Director%27s_Cut).webm from Gregory Wilson (Director and cinematographer), CC BY 3.0 (https://creativecommons.org/licenses/by/3.0), via Wikimedia Commons.

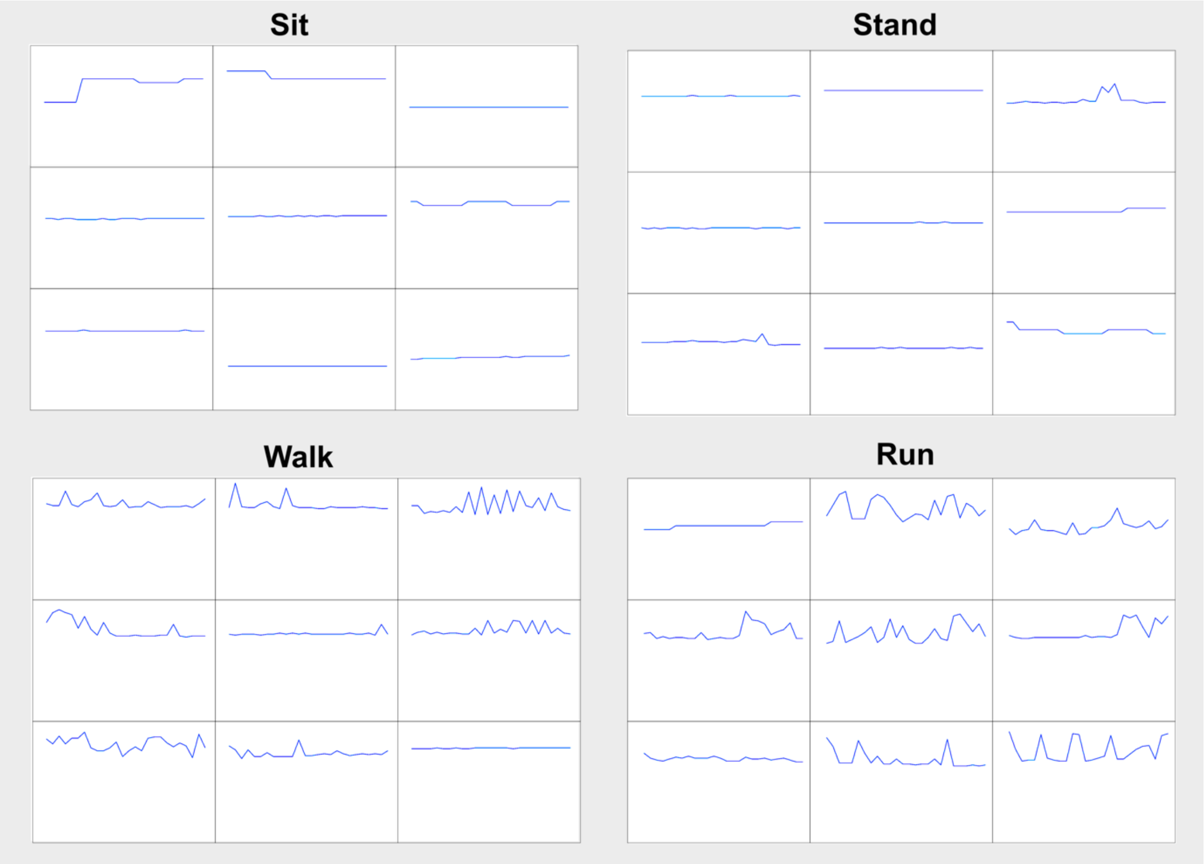

Supplementary Fig. 10: Examples of temperature-time trends for the different activities. Each box contains a 12 s temperature-time trend.

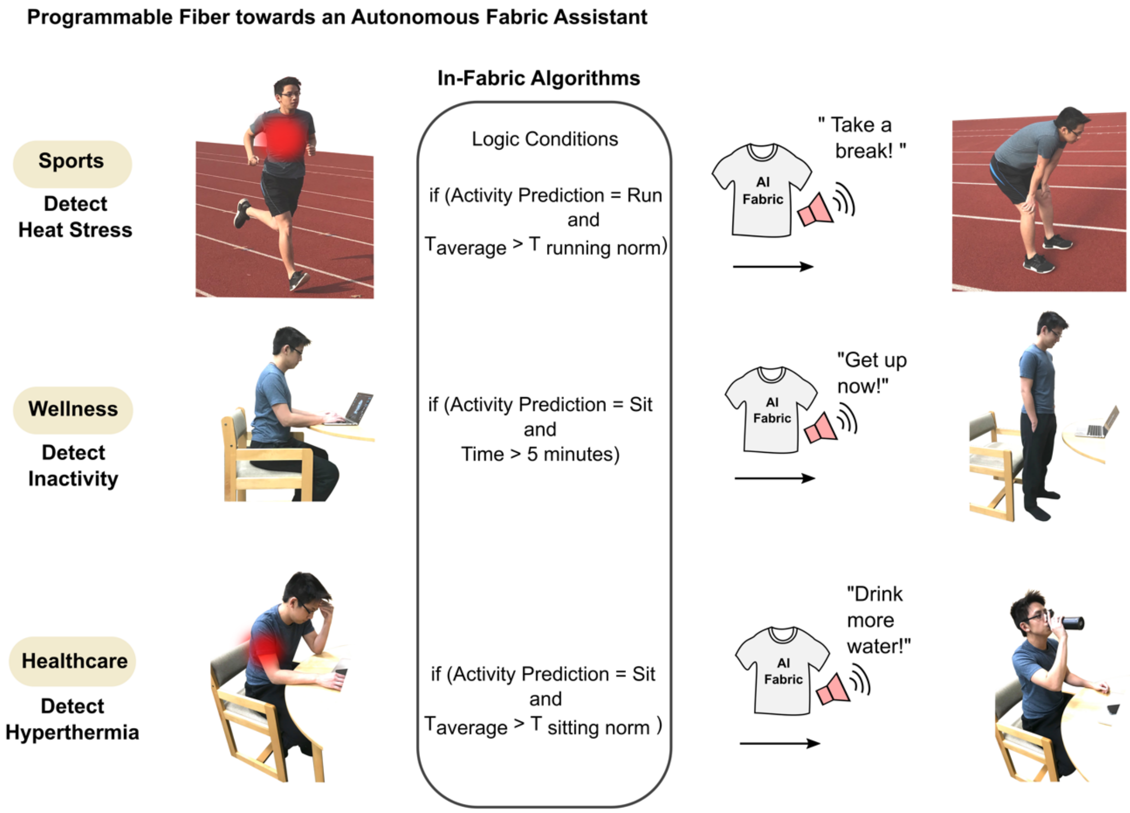

Supplementary Fig. 11: Examples of applications of the digital fibre programmed with different algorithms to enable an autonomous fabric-based personal assistant for sports, wellness, and healthcare applications. In the first example,temperature tracking for sports, the fabric detects that the wearer is running via its human activity recognition algorithms, and starts analyzing the wearer's body temperature. Upon reading an average temperature which is higher than the normal running baseline indicating potential heat stress, the digital fabric, equipped with a small (diameter ~1 cm) speaker, prompts the wearer to take a break. In the second example, an algorithm for inactivity tracking is stored in the digital fibre. The fabric tracks inactivity for long durations and autonomously prompts the wearer with a voice command to stand. In the third example, an in-fabric algorithm tracks rest-state temperature to predict potential hyperthermia or fever. Upon recognizing that the wearer is sitting and sensing a body temperature higher than the normal rest-state, the fabric prompts the wearer to hydrate. The normal temperature for different activities is measured by averaging the multi-day data stored in the digital fibre. All voice commands are stored in the digital memory fibre.
