# Supplementary material for: Digital electronics in fibres enable fabric-based machine-learning inference
Source: Nat Commun. 2021 Jun 3;12:3317. doi: 10.1038/s41467-021-23628-5 (PMC8175338; doi:10.1038/s41467-021-23628-5)
Supplement: Supplementary file 3 — Description of Additional Supplementary Files [file 41467_2021_23628_MOESM3_ESM.docx]

**Supplementary Video 1**

Description: Fabrication Steps of Digital Fibers.
